# Reliability and validity of the Turkish version of the pain attention and awareness questionnaire for nonspecific musculoskeletal pain

**DOI:** 10.1186/s12891-026-09535-0

**Published:** 2026-01-23

**Authors:** Mahmut Sürmeli, Özlem Çinar Özdemir, Ceyhun Topcuoğlu

**Affiliations:** 1 Department of Physical Therapy and Rehabilitation, Tokat Gaziosmanpaşa University, Taşlıçiftlik Campus, Tokat, 60250 Türkiye; 2 Department of Physical Therapy and Rehabilitation, Bolu Abant İzzet Baysal University, Gölköy Campus, Bolu, 14030 Türkiye; 3 Department of Physical Therapy and Rehabilitation, Munzur University, Aktuluk Campus, Tunceli, 62000 Türkiye

**Keywords:** Cultural adaptation, Validation, Reliability, Psychometric properties, Pain

## Abstract

**Background:**

Reliable and valid assessments of pain vigilance and awareness are crucial for understanding and treating pain within cognitive-behavioral frameworks, such as the fear-Avoidance Model. While the Pain Vigilance and Awareness Questionnaire (PVAQ) is a widely used tool for this purpose, no culturally adapted and validated version exists for the Turkish-speaking population, creating an instrumental and clinical gap.

**Objective:**

This study aimed to translate, culturally adapt, and comprehensively evaluate the psychometric properties of the Turkish version of the PVAQ in a sample of patients with nonspecific musculoskeletal pain.

**Methods:**

An instrumental, cross-sectional study was conducted. The PVAQ was translated and adapted into Turkish according to established guidelines. A sample of 131 patients with low back, neck, or shoulder pain completed the PVAQ-T, the Pain Catastrophizing Scale (PCS), the Tampa Scale for Kinesiophobia (TSK), the Hospital Anxiety and Depression Scale (HADS), and a visual analog scale (VAS) for pain intensity. Reliability was assessed via internal consistency (Cronbach’s alpha) and test-retest reliability (intraclass correlation coefficient - ICC). Construct validity was examined via exploratory factor analysis (EFA) and confirmatory factor analysis (CFA). Criterion validity was evaluated through Pearson correlations with the PCS, TSK, and HADS.

**Results:**

The PVAQ-T demonstrated acceptable internal consistency (Cronbach’s α = 0.83) and good test-retest reliability (ICC = 0.918). EFA supported a two-factor structure (vigilance and awareness), explaining 62.3% of the variance. CFA indicated that the original 16-item model had a suboptimal fit (CFI = 0.807; GFI = 0.732; SRMR = 0.060; RMSEA = 0.138, and χ²/df = 3.46). Following the removal of items 4 and 12 based on exploratory and item-level findings, some fit indices showed modest improvement (CFI = 0.836; GFI = 0.768; SRMR = 0.006), whereas others remained unchanged or worsened (RMSEA = 0.144; χ²/df = 3.69). The PVAQ-T total score showed moderately to strongly, statistically significant (*p* < 0.001) correlations with the PCS (*r* = 0.681), TSK (*r* = 0.694), HADS-Anxiety (*r* = 0.491), and HADS-Depression (*r* = 0.416) scores, supporting its criterion validity.

**Conclusion:**

Within a Turkish sample of individuals with nonspecific musculoskeletal pain, the 14-item Turkish version of the PVAQ demonstrates adequate reliability and validity for assessing pain-related attention and awareness. While the two-factor structure was supported at the exploratory level, confirmatory findings suggest that results should be interpreted with caution. The PVAQ-TR may be a useful instrument for research and clinical assessment of pain-related attentional processes, highlighting the importance of cultural adaptation and empirical evaluation of psychometric instruments.

**Supplementary Information:**

The online version contains supplementary material available at 10.1186/s12891-026-09535-0.

## Background

 Pain is a complex and multidimensional experience and represents a significant public health challenge [1]. According to the International Association for the Study of Pain, pain is defined as “an unpleasant sensory and emotional experience associated with, or resembling that associated with, actual or potential tissue damage.” Building on this broad definition, pain that lasts longer than three months is specifically classified as chronic pain in clinical and research contexts [2]. Unlike acute pain, which serves as a physiological warning sign, chronic pain is frequently associated with functional limitations, psychological distress, and altered pain-related cognitions, highlighting its distinct etiological and psychosocial complexities [2, 3].

Recent global evidence has demonstrated that musculoskeletal pain is highly prevalent across diverse populations and countries, affecting a substantial proportion of individuals in both rural and urban settings. These findings highlight the global burden of musculoskeletal pain and its relevance across different sociocultural and healthcare contexts [4]. Similarly, musculoskeletal pain represents a significant health concern in Turkey as well. According to data from the Turkish Statistical Institute in 2022, back pain was reported as the most common health complaint, followed by neck pain, over the preceding 12 months. Approximately 24.6% of individuals experienced low back pain, while 17.2% reported neck pain. These findings highlight the substantial burden of musculoskeletal pain within the general population and underline the need for valid and reliable assessment tools targeting pain-related cognitive and attentional processes [5].

Pain experience extends beyond mere nociception, and is shaped by diverse factors, such as social context, emotion, attentional focus, and perception, which leads to individual differences in pain behavior and perception [6]. In particular, processes based on previous experiences play an important role in the experience of pain, pain behavior, and pain related responses [7]. In this context, processes such as caution, attention, catastrophizing, or focusing on pain play critical roles in understanding the pathogenesis and maintenance of chronic pain [8, 9].

Excessive attention to pain is considered a central component in etiological models such as the fear-avoidance model [10, 11]. According to this model, individuals who interpret pain as threatening develop pain fear, which leads to increased attention to pain (caution, hypervigilance) and avoidance behaviors [11]. Over time, this process can result in physical deactivation, depression, and chronic pain [12]. Consequently, the reliable and valid assessment of pain vigilance is of considerable importance for both clinical evaluation and research, particularly in understanding pain-related cognitive mechanisms and monitoring changes over time [13].

Pain vigilance refers to an individual’s attention to pain-related stimuli and can be conceptualized as “excessive focus on pain” [8, 13]. Considering the effects of pain on individuals’ social, psychological, and physical conditions, their focus on pain becomes clinically important. The Pain Vigilance and Awareness Questionnaire (PVAQ) was developed to assess the state of vigilance and awareness in pain. The initial psychometric properties of the PVAQ were evaluated by McCracken in 80 patients with low back pain and demonstrated good internal consistency (Cronbach’s α = 0.86) and adequate reliability (*r* = 0.80) [13]. Furthermore, PVAQ scores were shown to be positively correlated with personal body awareness and negatively correlated with pain neglect, indicating construct validity [8, 13].

The psychometric evaluation of the PVAQ has demonstrated strong reliability and construct validity in various studies [14–21]. Studies have confirmed the two-factor structure of the questionnaire, and responses have been divided into two categories: “attention to pain” and “attention to changes in pain” [8, 15, 16, 20]. These categories can also be referred to as “active alertness” and “passive awareness,” reflecting different dimensions of pain-related attention [16]. Furthermore, the PVAQ has demonstrated good psychometric properties in different populations, including individuals with chronic pain conditions and healthy individuals. In studies conducted with fibromyalgia patients, the Spanish version of the PVAQ provided satisfactory reliability and validity, confirming its suitability for use in translational contexts [15]. These robust psychometric properties demonstrate the questionnaire’s effectiveness in assessing pain awareness for both clinical and research applications [15, 18, 21, 22].

Despite the high prevalence of pain, there is no measurement tool that can assess pain vigilance, is adapted to Turkish culture, and is valid and reliable. This deficiency limits both clinicians and researchers in objectively evaluating the cognitive processes associated with pain. The translation of the questionnaire into Turkish will enable the examination of the relationships between pain focus and individuals’ social, psychological, and physical experiences. This will increase the importance of cognitive and behavioral approaches alongside pharmacological methods among treatment options related to pain in clinical practice. Therefore, the aim of this study was to adapt the PVAQ into Turkish and examine its validity and reliability in a sample of patients with nonspecific musculoskeletal pain. This study is expected to contribute to pain research and clinical practice in Turkey and to provide an important tool for targeting and evaluating the effectiveness of cognitive-behavioral interventions in pain management.

## Methods

### Study design and participants

This study is an instrumental [23], cross-sectional study conducted for the Turkish adaptation and psychometric evaluation of the PVAQ.

The sample size was determined on the basis of the number of items in the scale, taking into account the rule that at least 10 participants should be included for each item [24]. Initially, 160 participants were recruited for the study. Following the exclusion of participants with incomplete questionnaires or missing responses exceeding the predefined criteria, data from 131 participants who completed both assessments were included in the final analysis.

The study included individuals aged 20–70 years who had nonspecific back, neck, or shoulder pain lasting at least 1 month who voluntarily agreed to participate in the study, provided informed consent, were literate, and had the cognitive ability to understand the questionnaire. Participants’ cognitive ability to understand and respond to the questionnaire was determined through a brief structured face-to-face interview conducted by the researcher prior to data collection. Individuals who were able to comprehend the study instructions, provide informed consent, and respond coherently to the questionnaire items were considered cognitively eligible.

Individuals with pain in other parts of the body, whose primary cause of pain was related to a specific medical condition (e.g., trauma, post-surgery, inflammatory diseases), who had cognitive/mental developmental delays that could cause difficulty in understanding the questionnaire, who had arthritis, cancer, osteoporosis, or chronic inflammatory diseases, psychiatric diagnoses, or serious psychological disorders that could alter the pain experience, and who had recently undergone surgical, pharmacological, or invasive procedures for pain treatment were excluded from the study. Exclusion criteria were evaluated based on self-reported medical history, researcher-administered screening questions, and a brief clinical interview conducted before questionnaire administration. Participants who met any of the exclusion criteria were not included in the study.

The study recruited outpatients with nonspecific low back, neck, or back pain through community outreach and social media announcements. The participants who completed the initial assessment provided their contact information, which was subsequently used to invite them for the follow-up evaluation. The study protocol was approved by the Munzur University Noninterventional Research Ethics Committee (Approval No: 2025-08), and all participants provided verbal and written informed consent. Clinical trial number: not applicable.

### Language adaptation

The linguistic and cultural adaptation of the questionnaire was carried out in accordance with protocols appropriate for the research objective and design, and in line with the best practice principles for patient-reported outcome measures [25].

### Content validity

The items from the original questionnaire were translated into Turkish by bilingual translators whose native language is English and who also know Turkish, with the aim of preserving the conceptual meaning and using culturally and clinically appropriate expressions. Detailed explanations were provided to the translators to ensure that they fully understood the concepts in the PVAQ, thereby preserving the conceptual meaning. Two professional translators subsequently independently translated the text into everyday language. Differences between the translators with minor wording adjustments were made to improve clarity and cultural appropriateness, while preserving the original conceptual meaning of the items. The resulting Turkish draft was then independently back-translated into English. The researchers compared the back-translations with the items of the original scale and, together with the translators, ensured that the Turkish version reflected the original content and conceptual equivalence. The expert committee did not identify any items requiring deletion.

For adaptability and appropriateness to Turkish society, the translations were submitted to an expert committee consisting of bilingual clinicians. The committee examined the semantic, idiomatic, and conceptual equivalence of the items and responses, identifying any inconsistencies or errors. Content validity was evaluated qualitatively through expert consensus focusing on semantic, idiomatic, and conceptual equivalence. Quantitative content validity indices (e.g., Aiken’s V or Kappa) were not calculated, as the primary aim at this stage was to ensure conceptual and cultural appropriateness rather than item-level quantitative agreement.

### Pilot test

The final version of the translation was administered to 20 patients with low back pain to assess its comprehensibility and cognitive equivalence. Cognitive interviews were conducted to identify conceptually inappropriate items and elements that could cause confusion, and the final version of the scale was developed. To assess test–retest reliability, the second administration of the questionnaire was conducted 10 days after the first assessment to prevent participants from recalling their initial responses and to minimize the risk of changes in their clinical status. During the pilot testing, no substantial comprehension difficulties were identified. Based on participant feedback, no structural changes to the questionnaire were required, and the final Turkish version was confirmed for psychometric testing.

### Data collection instruments

The physical characteristics of the participants who agreed to take part in the study, including age (years), height (meters), and body weight (kilograms), as well as sociodemographic data such as educational status and occupation, were recorded on a data collection form specifically designed for the study. In addition, clinical characteristics such as pain intensity, location, duration, previous and current treatments, medication use, and medical history were also documented.

#### The pain vigilance and awareness questionnaire (PVAQ)

It was used to assess the frequency of individuals’ habitual attention to pain by evaluating their self-reported monitoring of pain and changes in pain over the past two weeks. The original language of the PVAQ is English. The questionnaire consists of 16 items, each rated on a Likert scale ranging from 0 to 5, where 0 corresponds to “Never,” 1 to “Rarely,” 2 to “Sometimes,” 3 to “Often,” 4 to “Almost always,” and 5 to “Always.” Participants are asked to select the option that best reflects their behavior over the past two weeks. The responses to items 8 and 16 are reverse scored, such that “Never” equals 5 points and “Always” equals 0 points. Items 1, 6, 7, 8, 10, 12, 13, 14, 15, and 16 assess attention to pain, whereas items 2, 3, 4, 5, 9, and 11 measure awareness of changes in pain. The total score is obtained by summing the item scores, resulting in a range from 0 to 80. Higher scores indicate greater vigilance or attentiveness to pain—in other words, a higher level of pain awareness [13]. To assess the concurrent validity of the PVAQ, participants also completed the questionnaire listed below:

#### Pain catastrophizing scale (PCS)

This scale consists of 13 items and assesses the dimension of catastrophizing (exaggeration) of pain in individuals with musculoskeletal complaints or other conditions. Each question in the questionnaire is scored from “0” (never) to “4” (always). The total score ranges from 0 to 52 [26]. The Turkish version of the questionnaire was used. It has demonstrated excellent internal consistency (Cronbach’s α = 0.95), good test–retest reliability (ICC = 0.83), and confirmed construct validity through confirmatory factor analysis in individuals with chronic musculoskeletal pain [27].

#### Tampa scale of kinesiophobia (TSK)

This scale consists of 17 items and measures fear and avoidance behaviors related to pain. The questionnaire uses a 4-point Likert scale ranging from “1” (strongly disagree) to “4” (strongly agree). After items 4, 8, 12, and 16 are reversed, a total score is calculated. The total score ranges from 17 to 68, with higher scores indicating higher levels of kinesiophobia. The Turkish version of the Tampa Scale for Kinesiophobia has demonstrated excellent test–retest reliability (ICC = 0.81, 95% CI = 0.72–0.87) in individuals with neck and low back pain and has been shown to be suitable for use in clinical settings [28].

#### Visual analog scale (VAS)

The VAS consists of a 10 cm straight line, where the left end corresponds to a score of ‘0’ and the right end corresponds to ‘10’. The pain increases as one move from left to right along the line. The participants were instructed to mark the intensity of the pain they were currently experiencing on the 10 cm line, considering the left end as “0 – no pain at all” and the right end as “10 – the most severe, unbearable pain.” The distance from the ‘0’ point to the marked point was subsequently measured in centimeters (cm) via a tape measure, and the obtained value was recorded as the pain intensity score [29].

#### Hospital anxiety and depression scale (HADS)

This scale is used to screen for anxiety and depression in individuals with physical illness [30]. The scale consists of a total of 14 items, seven of which assess depression and seven of which assess anxiety, and each item is scored from 0 to 3. High scores indicate increased levels of anxiety and depression. A Turkish validated version of the scale was used. It has demonstrated good internal consistency, with Cronbach’s alpha values of 0.85 for the anxiety subscale and 0.78 for the depression subscale. Construct validity was supported by a two-factor structure corresponding to anxiety and depression [31].

### Statistical analysis

The data obtained in the study were analyzed via IBM SPSS 27.0 Statistics. The means and standard deviations were calculated for continuous variables, whereas frequencies and percentages were reported for categorical variables. Item-level distributional properties were examined using skewness and kurtosis values, with values between − 2 and + 2 considered indicative of acceptable univariate normality [32]. The internal consistency of the scale was assessed via Cronbach’s alpha coefficient for both the total scale and each identified factor separately. To evaluate test–retest reliability, the intraclass correlation coefficient (ICC, 95% confidence interval) was calculated between the two administrations. To assess construct validity, the Kaiser–Meyer–Olkin (KMO) measure of sampling adequacy and Bartlett’s test of sphericity were first performed, with KMO values ≥ 0.60 considered acceptable and Bartlett’s test required to be statistically significant [33], followed by exploratory factor analysis (EFA). In the EFA, Principal Component Analysis (PCA) was utilized as the extraction factor to identify the underlying structure of the scale by maximizing the total variance explained. The factor structure obtained from the EFA was then tested via confirmatory factor analysis (CFA). The explained variance was reported for each factor individually and for the total scale. Model fit was evaluated using the following indices and criteria: χ²/df < 5, root mean square error of approximation (RMSEA) ≤ 0.08, standardized root mean square residual (SRMR) ≤ 0.08, and comparative fit index (CFI) and goodness-of-fit index (GFI) values ≥ 0.90, in accordance with established recommendations [34, 35]. For criterion validity, Pearson correlation analysis was conducted between the total scores of the PVAQ and those of the PCS, TSK, and HADS. A significance level of *p* < 0.05 was considered statistically significant in all analyses. To address the initial lack of model fit, overall model fit indices were evaluated, and item-level psychometric indicators—primarily based on exploratory factor analysis results and item–total correlations—were examined to inform decisions regarding item retention [36].

## Results

### Sample characteristics

A total of 131 individuals with musculoskeletal pain participated in the study. The mean age of the participants was 37.7 ± 11.9 years, and 52.7% were male. The majority of the sample reported low back pain (59.5%). The mean body mass index was 26.2 ± 4.6 kg/m². The average pain duration was 9.1 ± 12.1 months, and the mean pain intensity was 4.48 ± 1.58 on a 0–10 numerical rating scale. The details are presented in Table 1.

**Table 1:** Demographic and clinical characteristics of the participants

		Mean ± S.D.	Median (min - max)	*n*	%
Age (years)		37.74 ± 11.85	36 (22–72)	131	100
Height (cm)		167.2 ± 10.68	168 (142–194)	131	100
Body weight (kg)		72.95 ± 11.78	75 (48–105)	131	100
BMI (kg/m^2^)		26.22 ± 4.56	25.53 (18.21–40.68)	131	100
Pain duration (months)		9.14 ± 12.06	4 (1–84)	131	100
Pain intensity (0–10 scale)		4.48 ± 1.58	4 (1–8)	131	100
Sex	Male			69	52.7
	Female			62	47.3
Marital status	Single			46	35.1
	Married			77	58.8
	Widowed			8	6.1
Education level	Literate			6	4.6
	Primary school			13	9.9
	Middle school			17	13.0
	High school			24	18.3
	University			71	54.2
Pain location	Low back			78	59.5
	Neck			25	19.1
	Thoracic			4	3.1
	Low back & neck			4	3.1
	Low back & thoracic			8	6.1
	Neck & thoracic			9	6.9
	Low back, neck & thoracic			3	2.3

*BMI* Body mass index, *cm* centimeter, *kg* kilogram, *n* sample size

### Item-level descriptive statistics

Item-level descriptive statistics for the initial and retest administrations of the PVAQ, along with corrected item–total correlations and Cronbach’s alpha values if items were deleted, are presented in Table 2. Mean item scores at the initial assessment ranged from 1.79 ± 0.81 to 3.46 ± 0.79, while retest means ranged from 1.88 ± 0.81 to 3.22 ± 0.83, indicating a comparable distribution of responses across administrations.

Corrected item–total correlation coefficients varied across items, ranging from − 0.568 to 0.794. Lower item–total correlations were observed for Items 4 and 12. Cronbach’s alpha coefficients calculated if these items were deleted were higher than the overall alpha value. Item-level descriptive statistics supported further examination of the factor structure.

The mean total PVAQ score was 42.7 ± 6.2 at the initial assessment and 41.5 ± 6.2 at the retest. The similarity between the total scores across the two administrations indicates that the scale provides stable measurements over time. (Table 2)

**Table 2:** Descriptive statistics, item-total correlations, and internal consistency of the PVAQ: initial measurement and retest

Item	Mean ± SD (Initial Measurement)	Mean ± SD (Retest)	Corrected Item-Total Correlation	Cronbach’s α if Item Deleted
PVAQ 1	2.45 ± 0.83	2.34 ± 0.76	0.631	0.808
PVAQ 2	3.12 ± 0.74	2.99 ± 0.81	0.787	0.799
PVAQ 3	3.46 ± 0.79	3.22 ± 0.83	0.749	0.801
PVAQ 4	2.5 ± 0.61	2.52 ± 0.64	0.043	0.839
PVAQ 5	3.42 ± 0.81	3.18 ± 0.77	0.794	0.797
PVAQ 6	2.24 ± 0.86	2.3 ± 0.83	0.521	0.815
PVAQ 7	2.56 ± 0.66	2.44 ± 0.7	0.645	0.810
PVAQ 8	1.85 ± 0.78	1.88 ± 0.85	−0.439	0.869
PVAQ 9	3.41 ± 0.76	3.2 ± 0.76	0.785	0.799
PVAQ 10	3.11 ± 0.78	3.08 ± 0.79	0.623	0.809
PVAQ 11	3.07 ± 0.75	3.01 ± 0.7	0.534	0.815
PVAQ 12	2.92 ± 0.59	2.95 ± 0.68	0.465	0.820
PVAQ 13	2.42 ± 0.77	2.35 ± 0.84	0.648	0.807
PVAQ 14	2.47 ± 0.64	2.35 ± 0.64	0.584	0.814
PVAQ 15	1.79 ± 0.81	1.88 ± 0.81	0.655	0.806
PVAQ 16	2.09 ± 0.85	2.06 ± 0.87	−0.568	0.880
PVAQ Total	42.73 ± 6.16	41.46 ± 6.2		

*PVAQ* Pain vigilance and awareness questionnaire, *SD* Standard deviation

Item distribution characteristics of the PVAQ are presented in Table X. Item-level skewness values ranged from − 0.22 to 0.83, and kurtosis values ranged from − 0.48 to 2.92. Floor effects ranged between 0.76% and 41.22%, while ceiling effects ranged between 0.76% and 8.40% across items (Table 3).

**Table 3:** Item distribution characteristics of the PVAQ (Initial Administration)

Item	Mean ± SD	Skewness	Kurtosis	Floor effect (%)	Ceiling effect(%)
PVAQ1	2.45 ± 0.83	0.68	0.36	7.63	1.53
PVAQ2	3.12 ± 0.74	0.14	−0.48	19.85	2.29
PVAQ3	3.46 ± 0.79	0.04	−0.42	9.92	8.40
PVAQ4	2.5 ± 0.61	−0.01	−0.34	3.05	3.05
PVAQ5	3.42 ± 0.81	−0.21	0.25	1.53	7.63
PVAQ6	2.24 ± 0.86	0.24	−0.25	20.61	0.76
PVAQ7	2.56 ± 0.66	0.44	−0.38	1.53	7.63
PVAQ8	1.85 ± 0.78	0.06	0.10	3.05	1.53
PVAQ9	3.41 ± 0.76	−0.22	0.61	1.53	6.11
PVAQ10	3.11 ± 0.78	−0.19	−0.29	1.53	1.53
PVAQ11	3.07 ± 0.75	0.11	−0.07	0.76	2.29
PVAQ12	2.92 ± 0.59	0.02	2.92	1.53	1.53
PVAQ13	2.42 ± 0.77	0.22	−0.32	9.16	8.40
PVAQ14	2.47 ± 0.64	0.28	−0.21	3.05	4.58
PVAQ15	1.79 ± 0.81	0.83	0.16	41.22	3.82
PVAQ16	2.09 ± 0.85	−0.18	−0.20	3.05	3.05

*PVAQ* Pain vigilance and awareness questionnaire, *SD* Standard deviation

### Internal consistency and test–retest reliability

The internal consistency of the total PVAQ score was Cronbach’s α = 0.83. At the subscale level, Cronbach’s alpha coefficients were 0.89 for the Vigilance factor and 0.88 for the Awareness factor. Internal consistency values for the total scale and subscales are presented in Table 4.

**Table 4:** Internal consistency and construct validity of the PVAQ (14-item version)

Factors	Number of Items	Explained Variance (%)	Cronbach’s α
Factor 1: Vigilance	8	54.50%	0.897
Factor 2: Awareness	6	10.69%	0.883
TOTAL	14	65.19%	0.934

Test–retest reliability was assessed using the intraclass correlation coefficient (ICC). The ICC for the total PVAQ score was 0.918 (95% CI: 0.884–0.942). Item-level ICC values ranged from 0.705 to 0.869 (Table 5).

**Table 5:** Reliability analyses of the Turkish version of the PVAQ (Cronbach’s α and test–retest ICC Values)

Item	ICC	95% CI Lower Bound		95% CI Upper Bound
PVAQ 1	0.869	0.815		0.907
PVAQ 2	0.834	0.765		0.882
PVAQ 3	0.811	0.734		0.866
PVAQ 4	0.799	0.716		0.858
PVAQ 5	0.809	0.731		0.865
PVAQ 6	0.849	0.787		0.893
PVAQ 7	0.794	0.708		0.854
PVAQ 8	0.842	0.777		0.888
PVAQ 9	0.829	0.758		0.879
PVAQ 10	0.832	0.763		0.881
PVAQ 11	0.776	0.684		0.842
PVAQ 12	0.705	0.583		0.791
PVAQ 13	0.857	0.799		0.899
PVAQ 14	0.739	0.632		0.815
PVAQ 15	0.845	0.781		0.890
PVAQ 16	0.865	0.809		0.904
PVAQ Total	0.918	0.884		0.942
Analysis			Result	
Cronbach’s α			0.830	

*PVAQ* Pain vigilance and awareness questionnaire, *ICC* Intraclass correlation coefficient, *CI* Confidence interval

### Exploratory factor analysis (EFA)

The suitability of the data for factor analysis was confirmed by a Kaiser–Meyer–Olkin (KMO) value of 0.90 and a statistically significant Bartlett’s test of sphericity (χ² = 1364.563, *p* < 0.001). Exploratory factor analysis was conducted using principal component analysis with varimax rotation.

A two-factor structure with eigenvalues greater than 1 was identified, accounting for 62.3% of the total variance. Factor loadings ranged from 0.50 to 0.83. The two factors corresponded to the Vigilance and Awareness dimensions of the original scale. Factor loadings for the initial solution are presented in Table 6. Following the exclusion of Items 4 and 12, a revised 14-item solution explained 65.19% of the total variance. The Vigilance factor accounted for 54.50% of the variance, while the Awareness factor accounted for 10.69%.

**Table 6:** Results of the exploratory factor analysis

Item	Factor 1 (Vigilance)	Factor 2 (Awareness)	Factor 1 after removing 4 & 12	Factor 2 after removing 4 & 12
PVAQ1-5	0.824	0.209	0.915	
PVAQ1-2	−0.804	−0.041	0.788	
PVAQ1-3	−0.772	−0.228	0.881	
PVAQ1-15	0.709	0.397		−0.798
PVAQ1-13	0.688	0.423		−0.796
PVAQ1-9	0.635	0.452	0.862	
PVAQ1-1	0.632	0.424		−0.771
PVAQ1-7	0.521	0.452		−0.725
PVAQ1-6	−0.500	0.460	0.568	
PVAQ1-16	0.273	0.829		0.739
PVAQ1-14	0.383	0.795	0.604	
PVAQ1-10	0.422	0.714	0.638	
PVAQ1-8	0.443	0.713		0.652
PVAQ1-11	0.287	0.667	0.532	
PVAQ1-4	0.001	0.617		
PVAQ1-12	0.157	0.609		

*PVAQ* Pain Vigilance and Awareness Questionnaire, *PVAQ1* refers to the scores obtained from the first administration of the PVAQ

Dash (–) indicates that the item was not included in the factor after items 4 and 12 were removed

### Confirmatory factor analysis (CFA)

The CFA, conducted to test the fit of the original 16-item, two-factor model, yielded following fit indices: CFI = 0.807; GFI = 0.732; SRMR = 0.060; RMSEA = 0.138; χ²/sd = 3.46. Based on the EFA results and item-level psychometric findings, Items 4 and 12 were removed, and a revised 14-item model was tested. The revised model showed modest improvements in selected incremental fit indices (CFI = 0.836; GFI = 0.768; SRMR = 0.006), while absolute fit indices remained above recommended thresholds and RMSEA and χ²/df values increased slightly (RMSEA = 0.144; χ²/df = 3.69) (Table 7).

**Table 7:** Confirmatory factor analysis (CFA) results

Index	All items	Item 4 and 12 removed
CFI	0.807	0.836
GFI	0.732	0.768
SRMR	0.060	0.006
RMSEA	0.138	0.144
χ²	356.39	280.417
sd	103	76
χ²/df	3.46	3.69
p	0.0001*	0.0001*

*CFI* Comparative Fit Index, *GFI* Goodness-of-Fit Index, *SRMR* Standardized Root Mean Square Residual, *RMSEA* Root Mean Square Error of Approximation; *χ*² Chi-square, *df* degrees of freedom, *χ*²*/df* chi-square divided by degrees of freedom

^*^p = significance level

**Fig. 1:**
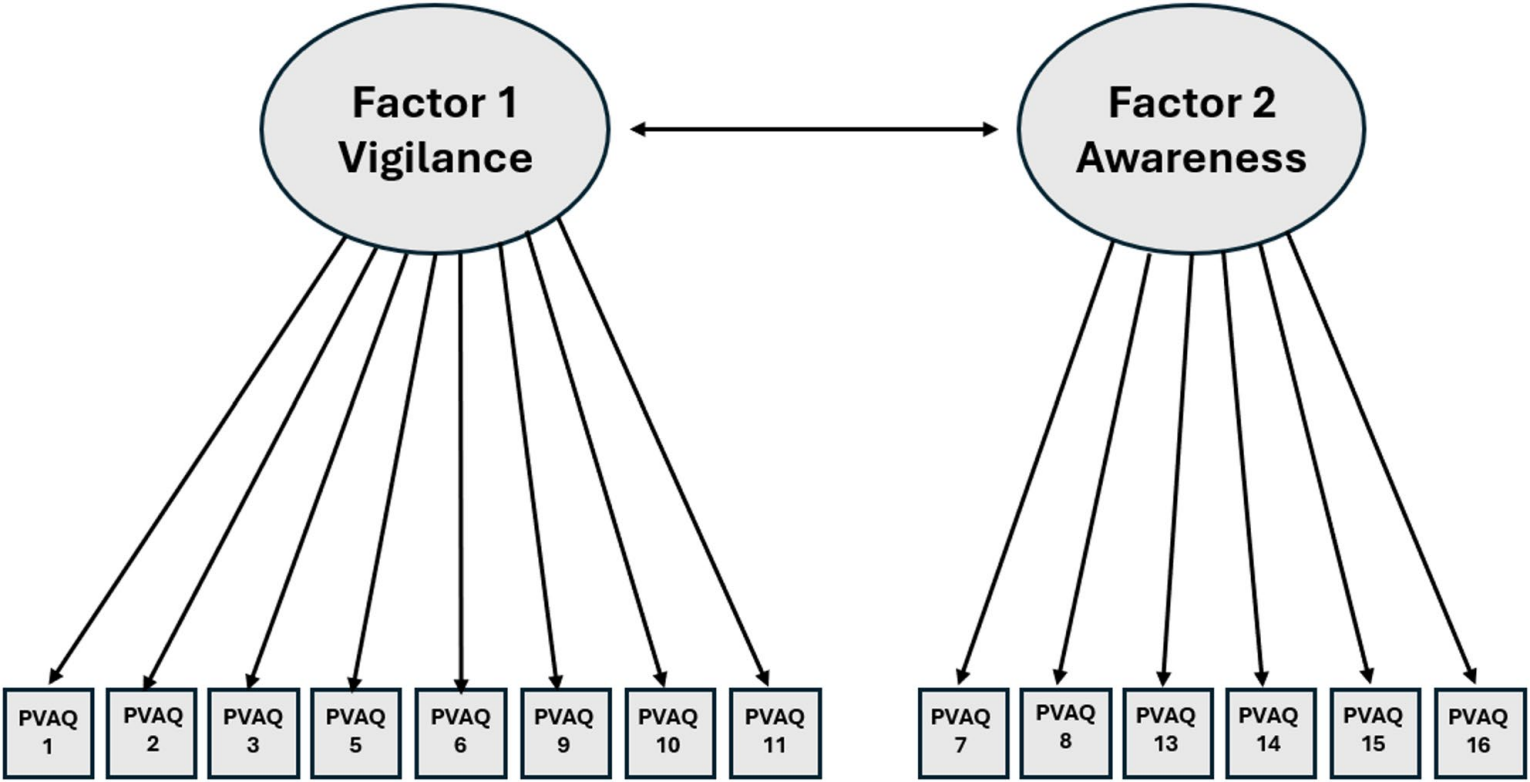
Diagram of the final two-factor confirmatory factor analysis model of the PVAQ-TR

The final two-factor CFA model of the PVAQ-TR is presented in Fig. 1.

### Validity analyses

Correlation analysis conducted to assess the criterion-related validity of the PVAQ revealed statistically significant (*p* < 0.001) positive correlations of moderate to strong strength between the total PVAQ scores and other scales. The strongest relationships were observed between the PVAQ and the PCS-Total (*r* = 0.681) and the TSK (*r* = 0.694). Significant correlations were also found with HADS-Anxiety (*r* = 0.491) and HADS-Depression (*r* = 0.416) (Table 8).

**Table 8:** Correlations between the PVAQ and other scales

Questionnaire		PVAQ 1	PVAQ 2
PCS-Total score	r	0.681	0.644
	p	*p* < 0.001*	*p* < 0.001*
PCS-R	r	0.642	0.606
	p	*p* < 0.001*	*p* < 0.001*
PCS-M	r	0.642	0.566
	p	*p* < 0.001*	*p* < 0.001*
PCS-H	r	0.626	0.598
	p	*p* < 0.001*	*p* < 0.001*
TKS	r	0.694	0.588
	p	*p* < 0.001*	*p* < 0.001*
HADS-Total score	r	0.485	0.401
	p	*p* < 0.001*	*p* < 0.001*
HADS-Anxiety	r	0.491	0.404
	p	*p* < 0.001*	*p* < 0.001*
HADS-Depression	r	0.416	0.348
	p	*p* < 0.001*	*p* < 0.001*

*PVAQ 1* First administration of the Pain Vigilance and Awareness Questionnaire, *PVAQ 2* retest administration, *PCS* Pain Catastrophizing Scale, *PCS-R* Rumination subscale, *PCS-M* Magnification subscale, *PCS-H* Helplessness subscale, *TKS* Tampa Kinesiophobia Scale, *HADS* Hospital Anxiety and Depression Scale, *HADS-Anxiety* Anxiety subscale, *HADS-Depression* Depression subscale, *HADS-Total* Total score

All correlations are reported as Pearson’s r with significance levels (*p < 0.001)

## Discussion

In this study, the validity and reliability of the Turkish version of the PVAQ were examined in individuals with nonspecific musculoskeletal pain. The findings provide evidence supporting adequate psychometric performance, particularly in terms of internal consistency, temporal stability, and convergent validity within this population. However, the results should be interpreted with consideration of the structural findings obtained from factor analyses. The factor analyses confirmed a two-factor structure (vigilance and awareness), which was consistent with the original form of the questionnaire. Furthermore, the significant associations observed between PVAQ scores and pain catastrophizing, kinesiophobia, disability, anxiety, and depression support the scale’s criterion-related validity. These results suggest that the Turkish version of the PVAQ can serve as a potentially useful assessment tool in research and supportive clinical assessment settings.

### Evaluation of reliability findings

The Turkish version of the PVAQ demonstrated adequate internal consistency, with a Cronbach’s alpha of 0.83 for the total scale. This finding aligns with the results reported in both the original study [8, 13] and other language adaptation studies of the PVAQ [8, 14–16, 18, 20], which reported alpha values ranging from acceptable to high levels across different populations and languages. These results indicate that the PVAQ-TR items have the potential to adequately and consistently measure aspects related to pain vigilance and awareness for this sample.

Additionally, test–retest reliability analysis revealed a high intraclass correlation coefficient over a 10-day interval, indicating that the PVAQ-TR yields stable scores over time in individuals with nonspecific musculoskeletal pain (0.918; 95% CI: 0.884–0.942). This level of temporal stability supports the use of the instrument in both clinical follow-up and research contexts where repeated assessments are required. Importantly, these findings were interpreted cautiously, without inferring responsiveness or sensitivity to change, which were beyond the scope of the present study.

### Evaluation of psychometric properties

The construct validity findings of the current study provide partial support for the theoretically confirmed two-factor structure of the PVAQ, including vigilance and awareness (attention to changes) dimensions. These findings are consistent with the theoretical framework initially described by Roelofs et al. [8] and subsequently replicated in international studies [16, 20]. These results support the view that pain attention encompasses not only active monitoring of pain sensations but also a passive awareness of subtle changes in the pain experience or bodily sensations.

However, the results of the CFA did not fully support the original 16-item model, and overall model fit indices remained below recommended thresholds (CFI = 0,807; GFI = 0,732; RMSEA = 0,138). Based on the EFA results item-level psychometric findings, items 4 and 12 were removed due to their relatively weak factor loadings and lower item–total correlations. These findings suggest that the conceptual equivalence or cultural interpretation of these items within the Turkish pain population may differs slightly from the original structure. Importantly, the decision to remove these items was driven primarily by EFA-based empirical evidence, rather than CFA modification indices.

Following item removal, the revised 14-item model demonstrated modest improvements in some incremental fit indices, while absolute fit indices remained above recommended thresholds. According to this pattern, the 14-item model does not clearly outperform the original structure in all absolute fit parameters. Although the two-factor structure is conceptually preserved, the CFA findings indicate that the structural validity of the scale should be interpreted with caution. Consequently, rather than viewing the 14-item version as an optimal model, it should be interpreted as providing adequate psychometric properties within the limitations of the current sample.

The decision to remove items 4 and 12 was informed not only by empirical findings but also by conceptual and interpretive considerations. Within the original conceptualization of the PVAQ, awareness is defined as a passive, non-evaluative monitoring of bodily sensations, whereas vigilance reflects a more active and evaluative attentional focus on pain-related information [8, 13]. During the review process, it was noted that items 4 (“I am quick to notice effects of medication on pain”) and 12 (“I seem to be more conscious of pain than others”) may invite interpretations that extend beyond passive monitoring of bodily sensations. Item 4 may be interpreted as reflecting an active and effortful attentional focus on detecting specific pain-related events, whereas Item 12 introduces a comparative self-evaluative judgment (“than others”), shifting attention toward appraisal-based evaluation of pain experience. Both characteristics may overlap with vigilance-related or evaluative attentional processes rather than the intended awareness dimension of the PVAQ. Such conceptual ambiguities may contribute to inconsistent interpretation among respondents [37] and weaken the conceptual coherence of the awareness subscale. Previous work on cross-cultural adaptation has demonstrated that subtle linguistic differences may alter the cognitive framing of items and weaken conceptual equivalence despite accurate translation [25]. In line with theoretical models of pain-related attention, attentional processes become maladaptive primarily when pain-related information is appraised as threatening rather than neutrally monitored [11, 38]. These issues have also been reported across cultures, as demonstrated in previous PVAQ adaptation studies, particularly within the awareness dimension [14, 15]. Consistent with these considerations, the weaker item-level performance observed for items 4 and 12 in the present study may reflect reduced conceptual alignment with the intended awareness construct.

The cumulative evidence, including poor item-level statistics, exploratory factor analysis results, and conceptual considerations, suggests that these two items should be removed for the Turkish version; however, this decision should be understood as contributing primarily to conceptual clarity and item-level coherence rather than indicating a substantial improvement in overall internal consistency. These findings aligns with other studies indicating that the factor structure of the PVAQ may vary across cultures, such as the modification of reverse-coded items in the Swedish adaptation [15] and the removal of seven items in the Spanish adaptation [14]. A possible explanation is that the factor structure of the PVAQ may be sensitive to cultural and linguistic context and underscores the importance of empirical evaluation in each adaptation study. Taken together, the 14-item structure may be considered a an empirically informed alternative for the Turkish sample, allowing for the exclusion of items with limited empirical contribution while maintaining the conceptual coherence of the awareness subscale.

Consequently, a 14-item Turkish version of the PVAQ may be considered within the context of the present study. This version, although longer than the Spanish and Swedish adaptations, appears to adequately maintain the conceptual integrity of the ‘awareness’ subscale. Nonetheless, a recurring observation across adaptation studies is that the original PVAQ’s factor structure is not universally replicated, highlighting the need to empirically determine the most suitable item set for each cultural context. Based on the current empirical findings, a 14-item configuration may be considered appropriate for the Turkish sample with nonspecific musculoskeletal pain, while acknowledging the need for further validation.

### Criterion validity and correlational findings

The correlations observed between the total scores of the PVAQ, and other psychological and functional assessment tools provide evidence supporting the criterion-related validity. These associations indicate that higher levels of pain vigilance and awareness are systematically related to established cognitive, emotional, and behavioral constructs relevant to the pain experience, thereby contributing to the construct validity of the PVAQ-TR.

Similarly, the strong positive correlation between the PVAQ and PCS scores suggests that pain-related cognitive processes, including heightened attention to pain, exaggeration of pain-related thoughts, feelings of helplessness, and rumination, are closely interconnected. This finding is consistent with theoretical frameworks describing maladaptive cognitive–emotional responses to pain, such as the fear-avoidance model, and supports the notion that increased attentional focus on pain may be associated with greater perceived threat and distress through catastrophizing processes [11]. These results are in agreement with those reported in the original PVAQ study [13] and the Italian adaptation by Monticone et al. [17]. Nevertheless, the comparatively weaker correlation observed in the Swedish fibromyalgia study [15] indicates that this relationship may differ across diagnostic populations and clinical contexts.

Similarly, the strong correlation between PVAQ and kinesiophobia is consistent with theoretical models suggesting that greater attention to pain is associated with increased perceptions of threat and avoidance-related behaviors. These findings position pain attention as a central trigger within the kinesiophobia–fear-avoidance cycle. In particular, the strong correlations with both pain catastrophizing and kinesiophobia underscore that hypervigilance to pain appears to be closely involved in the catastrophizing–fear of movement loop, as described in the fear-avoidance model [11].

In addition, significant correlations between PVAQ scores and symptoms of anxiety and depression indicate that pain vigilance is related to broader psychological distress in individuals with nonspecific musculoskeletal pain [39]. These findings suggest that sustained attentional focus on pain may be associated with emotional vulnerability and mood-related symptoms, underscoring the close interplay between pain-related cognitive processes and psychological comorbidities [19, 40]. These associations emphasize the multidimensional nature of chronic pain and the significance of considering emotional and cognitive factors alongside physical symptoms, not directly causal relationships.

Overall, the findings support the convergent validity of the Turkish version of the PVAQ, demonstrating that pain-related attention and awareness, as assessed by the scale, are meaningfully associated with relevant cognitive, emotional, and functional constructs within the Turkish population with nonspecific musculoskeletal pain. Despite potential differences in population and diagnosis, this pattern of associations is generally consistent with previous international validation studies.

### Clinical implications and recommendations

Clinical and research professionals may consider using a Turkish PVAQ version to measure pain awareness and attention in patients with nonspecific musculoskeletal pain. The instrument has the potential to identify patients with elevated levels of pain vigilance and to characterize the cognitive aspects of the pain experience. However, the PVAQ-TR should be utilized as a component of a multidimensional assessment framework rather than as a stand-alone measure for clinical decision-making or treatment evaluation due to the limitations noted in the confirmatory factor analysis.

### Limitations of the study

This study has several limitations. First, the sample size was low and both exploratory and confirmatory factor analyses were done in the same sample, which could hinder the generalizability of the structural findings. Second, it is imperative to exercise caution when extending the results to other pain populations due to the study’s exclusive focus on individuals with nonspecific musculoskeletal pain. Third, the confirmatory factor analysis yielded mixed fit indices even after item removal, and the decision to exclude items 4 and 12, while supported by item-level statistics and expert review, requires acknowledgment. The resulting 14-item PVAQ-TR model demonstrates adequate rather than optimal structural validity. This highlights the need for independent replication of the factor structure in future studies with larger Turkish samples to confirm its stability and generalizability. Finally, even though a 14-item version of the PVAQ-TR was proposed based on empirical findings, additional studies with independent samples are required to confirm its stability.

## Conclusion

For measuring pain-related attention and awareness in nonspecific musculoskeletal patients, the Turkish version of the Pain Vigilance and Awareness Questionnaire exhibits adequate reliability and validity. Confirmatory analyses suggested that the structural model should be interpreted cautiously, despite exploratory analyses supporting a two-factor structure that was consistent with the original conceptual model. The 14-item version, derived from empirical and conceptual considerations, may be considered for use with the awareness that its structural validity requires further confirmation. In general, the PVAQ-TR can serve as a useful tool for research and clinical settings that aim to comprehend the cognitive aspects of pain, when utilized with other assessment instruments.

## Supplementary Information

Supplementary Material 1.

## Data Availability

The datasets generated and/or analyzed during the current study are available from the corresponding author on reasonable request.

## Abbreviations

PVAQ: Pain Vigilance and Awareness Questionnaire
PVAQ-TR: Turkish version of the Pain Vigilance and Awareness Questionnaire
PCS: Pain Catastrophizing Scale
PCS-R: Pain Catastrophizing Scale – Rumination subscale
PCS-M: Pain Catastrophizing Scale – Magnification subscale
PCS-H: Pain Catastrophizing Scale – Helplessness subscale
TSK: Tampa Scale of Kinesiophobia
VAS: Visual Analog Scale
HADS: Hospital Anxiety and Depression Scale
HADS-A: Hospital Anxiety and Depression Scale – Anxiety subscale
HADS-D: Hospital Anxiety and Depression Scale – Depression subscale
EFA: Exploratory Factor Analysis
CFA: Confirmatory Factor Analysis
PCA: Principal Component Analysis
KMO: Kaiser–Meyer–Olkin measure of sampling adequacy
ICC: Intraclass Correlation Coefficient
CI: Confidence Interval
SD: Standard Deviation
BMI: Body Mass Index
RMSEA: Root Mean Square Error of Approximation
SRMR: Standardized Root Mean Square Residual
CFI: Comparative Fit Index
GFI: Goodness-of-Fit Index
χ²: Chi-square
df: Degrees of freedom
IASP: International Association for the Study of Pain
