# Supplementary material for: Reliability and validity of the Turkish version of the pain attention and awareness questionnaire for nonspecific musculoskeletal pain
Source: BMC Musculoskelet Disord. 2026 Jan 23;27:156. doi: 10.1186/s12891-026-09535-0 (PMC12914955; doi:10.1186/s12891-026-09535-0)
Supplement: Supplementary file 1 — Supplementary Material 1. [file 12891_2026_9535_MOESM1_ESM.docx]

**Appendix A. Translation and Back-Translation Procedure of the PVAQ**

| Step | Description |
| --- | --- |
| Forward translation | The original English version of the PVAQ was independently translated into Turkish by two bilingual translators with experience in health-related questionnaires. One translator had a clinical background, while the other focused on everyday language use to ensure clarity and cultural appropriateness. |
| Reconciliation | The two Turkish translations were compared item by item, and discrepancies were discussed by the translators and the research team until a single reconciled Turkish version was agreed upon. |
| Back-translation | The reconciled Turkish version was back-translated into English by an independent bilingual translator who was not involved in the initial translation process and was blinded to the original questionnaire. |
| Expert committee review | An expert committee consisting of bilingual clinicians reviewed all versions of the questionnaire. The committee evaluated semantic, idiomatic, and conceptual equivalence and suggested minor wording adjustments where necessary. |
| Pilot testing | The pre-final Turkish version was administered to 20 individuals with nonspecific musculoskeletal pain to assess item clarity, comprehensibility, and relevance. Participants were asked to comment on any items they found unclear or difficult to understand. |
| Finalization | Based on feedback from the pilot testing and expert review, minor revisions were made, and the final Turkish version of the PVAQ was established for psychometric testing. |
